# Foliar Glycine Betaine or Hydrogen Peroxide Sprays Ameliorate Waterlogging Stress in Cape Gooseberry

**DOI:** 10.3390/plants9050644

**Published:** 2020-05-19

**Authors:** Nicolas E. Castro-Duque, Cristhian C. Chávez-Arias, Hermann Restrepo-Díaz

**Affiliations:** Departamento de Agronomía, Facultad de Ciencias Agrarias, Universidad Nacional de Colombia, Bogotá 111321, Colombia; necastrod@unal.edu.co (N.E.C.-D.); hrestrepod@unal.edu.co (H.R.-D.)

**Keywords:** hypoxia, leaf gas exchange, waterlogging tolerance, organic compound, plant growth, *Physalis peruviana* L.

## Abstract

Exogenous glycine betaine (GB) or hydrogen peroxide (H_2_O_2_) application has not been explored to mitigate waterlogging stress in Andean fruit trees. The objective of this study was to evaluate foliar GB or H_2_O_2_ application on the physiological behavior of Cape gooseberry plants under waterlogging. Two separate experiments were carried out. In the first trial, the treatment groups were: (1) plants without waterlogging and with no foliar applications, (2) plants with waterlogging and without foliar applications, and (3) waterlogged plants with 25, 50, or 100 mM of H_2_O_2_ or GB, respectively. The treatments in the second trial were: (1) plants without waterlogging and with no foliar applications, (2) plants with waterlogging and without foliar applications, and (3) waterlogged plants with 100 mM of H_2_O_2_ or GB, respectively. In the first experiment, plants with waterlogging and with exogenous GB or H_2_O_2_ applications at a dose of 100 mM showed higher leaf water potential (−0.5 Mpa), dry weight (1.0 g), and stomatal conductance (95 mmol·m^−2^·s^−1^) values. In the second experiment, exogenously supplied GB or H_2_O_2_ also increased the relative growth rate, and leaf photosynthesis mitigating waterlogging stress. These results show that short-term GB or H_2_O_2_ supply can be a tool in managing waterlogging in Cape gooseberry.

## 1. Introduction

Cape gooseberry (*Physalis peruviana* L.) is a plant that belongs to the *Solanaceae* family and its center of origin is located in the Andes, specifically in Peru, from where it expanded to various areas of the tropics and subtropics [1,2,3]. In Colombia, the production of this crop was 16,445 t, occupying 1312 ha during 2018 [4].

Climate change and variability alter the normal rainfall cycle causing floods of agricultural land and affecting crop production [5]. In Colombia, climate variability phenomena, such as the “La Niña” phenomenon, are characterized by an increase in rainfall that enhances the probability of floods [6,7]. In 2010, La Niña phenomenon produced an increase in rainfall, exceeding historical averages and causing a decrease in agricultural production from 7888 to 1515 t in Cundinamarca, one of the main producer departments of the country [4,7,8].

It has been reported that there is a high susceptibility of cultivated plants to waterlogging stress, affecting their growth, development, yield, and finally their survival [9,10]. One of the main effects of waterlogging is on plant growth. In this regard, several authors have observed that moderate or prolonged periods of O_2_ deficit in the soil cause a low leaf area [11], a reduction in plant height [12], and an alteration in stem diameter [13]. In Cape gooseberry, short periods of waterlogging stress (6 days) also cause a decrease in plant height, leaf area, and stem diameter [14,15].

A reduction of growth parameters due to waterlogging may be associated with an impairment of the leaf gas exchange properties (stomatal conductance), chlorophyll content, and efficiency of photosystem II (PSII) [11,16,17,18]. Plants susceptible to waterlogging have been reported to show stomatal closure 24 h after the exposure to stress [19]. On the other hand, the leaf chlorophyll content can drop due to imbalances in the nutrient uptake or increased ethylene synthesis, causing impairment or decrease in the efficiency of PSII [19,20,21]. A previous experiment has also shown low stomatal conductance, leaf chlorophyll content, and *F_v_/F_m_* ratio in Cape gooseberry plants under moderate waterlogging periods (4 days) [15].

Waterlogging alters the plant water status due to stomatal closure [22]. The negative effects of periods of oxygen deprivation on the leaf water potential have been reported in cacao [17], bean [16], and tomato [23]. Likewise, the relative water content (RWC) has been widely used to describe the plant water status and has been correlated with the level of soil moisture [24]. In this regard, the RWC is a reliable variable to measure the susceptibility of plants to waterlogging [17,18]. These variables have also been useful to evaluate the susceptibility or efficiency of management techniques to O_2_ deficit conditions in the soil in Andean fruit trees such as Lulo or Cape gooseberry [15,20,25].

Exogenous applications of compounds such as glycine betaine (GB) or hydrogen peroxide (H_2_O_2_) can help tolerate or lessen negative effects on plants under abiotic stress conditions by activating defense mechanisms or aiding plant growth, development, and productivity [26,27]. Some authors have reported that physiological parameters such as leaf gas exchange properties (photosynthesis), efficiency of PSII, water relations (water potential), growth, and antioxidant activity are favored by these compounds under waterlogging stress in different cultivated species [13,28]. Glycine betaine helps plants under abiotic stress conditions by acting as an osmolyte that protects cells [29], increases cell water retention [30], reduces levels of reactive oxygen species (ROS) and helps in the protection of the plasma membrane [31]. Regarding waterlogging stress, the exogenous application of this molecule has been little studied; however, Rasheed et al. [28] reported that GB applications caused an increase in plant biomass, leaf total chlorophyll, and K^+^ concentration compared to fully waterlogged plants.

Hydrogen peroxide is a molecule that has also been studied to mitigate the effects of abiotic stresses in crops such as potato [32], tomato [33], bean [34,35], rice [36], maize [37] and soybean [13]. Different studies have concluded that exogenous H_2_O_2_ application helps leaf gas exchange properties (stomatal conductance and photosynthesis) [13], dry matter accumulation [35], leaf relative water content, and water potential [33,34], and plant height under different abiotic stresses [34,35]. Finally, the use of H_2_O_2_ has been little studied under waterlogging conditions. However, Andrade et al. [13] reported that pretreatments with H_2_O_2_ favored the increase in plant biomass, stomatal conductance, and net photosynthetic rate in soybean.

Increases in the intensity and frequency of rainfall in Colombia are estimated for the coming years [6,38]. For this reason, studies on the acclimatization response of Andean fruit trees to waterlogging scenarios have recently gained importance [14,20,21]. However, research on agronomic strategies to mitigate the negative impact of waterlogging with foliar GB and H_2_O_2_ sprays on Andean fruit trees has yet to be explored. Rasheed et al. [28] and Andrade et al. [13] mention the positive effect of these molecules on tolerance to waterlogging stress. For this reason, the objective of this study was to evaluate the exogenous application of different doses of GB or H_2_O_2_ on the physiological behavior of Cape gooseberry plants ecotype Colombia subjected to waterlogging, to determine the best molecule and dose to use to mitigate this stress.

## 2. Results

### 2.1. First Experiment: Evaluation of Different Doses of Glycine Betaine (GB) or Hydrogen Peroxide (H_2_O_2_) under a Waterlogging Period

**Figure 1:**
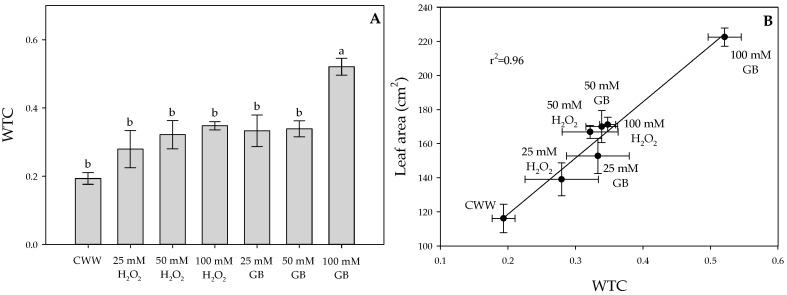
(**A**) Waterlogging Tolerance Coefficient (WTC) at 13 Days After Waterlogging (DAW) and (**B**) correlation between the leaf area and the WTC of Cape gooseberry (*Physalis peruviana* L.) plants ecotype Colombia subjected to a waterlogging period with treatments of control with waterlogging (CWW), 25 mM of hydrogen peroxide (H_2_O_2_), 50 mM of hydrogen peroxide (H_2_O_2_), 100 mM of hydrogen peroxide (H_2_O_2_), 25 mM of glycine betaine (GB), 50 mM of glycine betaine (GB) and 100 mM of glycine betaine (GB). Evaluated 13 days after waterlogging (DAW). Data represent the average of ten plants ± standard error per treatment (*n* = 5). Bars followed by different letters indicate statistically significant differences according to the Tukey test (*p* ≤ 0.05).

Table 1 summarizes the effect of foliar GB and H_2_O_2_ sprays on the growth parameters of Cape gooseberry plants. Control plants without waterlogging (CWoW) (not exposed to waterlogging) generally showed the highest growth parameter values throughout the experiment compared to the other treatments. In this sense, foliar GB or H_2_O_2_ sprays mainly contributed to a greater stem length in Cape gooseberry plants under waterlogging conditions at 4 Days After Waterlogging (DAW), with approximate stem length values of 21 cm, while plants with waterlogging and without any foliar compound sprays (control with waterlogging, CWW) had a height of 16.70 cm. At 4 DAW, it was also observed that the foliar applications of both compounds at their different doses favored the leaf area, stem diameter, and shoot dry weight of waterlogged plants. At 13 DAW, the obtained results of plant growth showed that foliar GB applications at a concentration of 100 mM caused an increase mainly on stem diameter (0.53 cm), leaf area (222.56 cm^2^), and shoot dry weight (1.06 g) in waterlogged plants compared to the CWW (0.42 cm, 116.14 cm^2^, and 0.39 g, respectively). Regarding foliar H_2_O_2_ applications, this compound directly affected the plant height (22.10 cm) of waterlogged plants, while the CWW showed values of 16.56 cm. Table 2 shows how foliar GB or H_2_O_2_ applications at their different doses influenced physiological variables such as leaf temperature, stomatal conductance (*g_s_*), efficiency of PSII (*F_v_/F_m_*), and water potential (*Ψ_wf_*) in Cape gooseberry leaves at 4 and 13 DAW, respectively. It is observed that waterlogging causes a higher leaf temperature (26.89 and 28.49 °C) and lower *g_s_* (157.10 and 42.76 mmol CO_2_·m^−2^·s^−1^) in plants at both sampling points. Foliar GB or H_2_O_2_ applications, mainly at a dose of 100 mM, caused a reduction in leaf temperature (22.27 and 24.71 °C, respectively) and an increase in *g_s_* (180.70 and 191.78 mmol CO_2_·m^−2^·s^−1^, respectively) at 4 DAW, with similar values to the ones recorded for plants without waterlogging (18.85 °C and 194.42 mmol CO_2_·m^−2^·s^−1^). Similar trends were also observed for the variables previously described at 13 DAW. On the other hand, the *F_v_/F_m_* ratio was also conditioned by the treatments at both points, with the lowest ratio being obtained in the CWW treatment (around 0.6). Furthermore, foliar applications of these compounds helped to increase this ratio (~0.77). Finally, the *Ψ_wf_* was higher in the control without waterlogging and in the GB treatment at a dose of 100 mM, compared to the other treatments in both samples. The Waterlogging Tolerance Coefficient (WTC) was obtained only at 13 DAW (Figure 1A), observing that the foliar GB application at 100 mM caused greater tolerance to waterlogging (0.52) compared to the rest of the treatments. Then, the correlation between leaf area and WTC (*r*^2^ = 0.96) also confirmed that the foliar GB or H_2_O_2_ sprays at a concentration of 100 mM were the best at conferring tolerance to a waterlogging condition (Figure 1B).

### 2.2. Experiment 2: Evaluation of the Most Efficient Doses of Glycine Betaine (GB) and Hydrogen Peroxide (H_2_O_2_) in Plants Exposed to Two Waterlogging Periods

Growth parameters (stem diameter, shoot dry weight, and leaf area) showed differences (*p* ≤ 0.05) between treatments throughout experiment 2 (Table 3). Regarding stem diameter, it was observed that foliar GB or H_2_O_2_ applications began to cause an increase in this variable under stress conditions from 4 DAW, maintaining this trend during the experiment. At the end of the trial (36 DAW), higher stem diameter (0.49 cm) values were observed in plants without waterlogging and with no foliar applications compared to waterlogged plants without any foliar application (0.31 cm). Exogenously supplied GB or H_2_O_2_ promoted an increase in stem diameter in waterlogged plants (0.39 cm for GB and 0.35 for H_2_O_2_). On the other hand, the shoot dry weight was considerably higher in plants without waterlogging (9.90 g) than in waterlogged plants with or without foliar applications (~1.6 g). Finally, foliar GB or H_2_O_2_ sprays caused an increase in leaf area (95.53 cm^2^ for GB and 91.54 cm^2^ for H_2_O_2_) compared to only waterlogged plants (25.82 cm^2^). However, plants under waterlogging with foliar applications did not reach the values obtained in plants without conditions of hypoxia in the soil or foliar sprays (1236.10 cm^2^) at the end of the trial (36 DAW).

The stomatal conductance (*g_s_*), leaf relative chlorophyll content (soil plant analysis development (SPAD) readings), and efficiency of PSII (*F_v_/F_m_*) were significantly affected by the treatments (*p* ≤ 0.05) (Table 4). Regarding *g_s_*, it is observed that the group of waterlogged plants with and without foliar applications of the compounds always showed lower values (between 9.90 and 40.63 mmol CO_2_·m^−2^·s^−1^) throughout the experiment (at 6, 12, 18, and 36 DAW, respectively) compared to control plants without waterlogging (CWoW) (between 118 and 216 mmol CO_2_·m^−2^·s^−1^ at the different sampling points). However, it was observed that GB or H_2_O_2_ sprays favored this variable in the group of waterlogged plants, observing significant differences (*p* ≤ 0.05) (31.78 and 40.63 mmol CO_2_·m^−2^·s^−1^, respectively) compared to CWW (9.90 mmol CO_2_·m^−2^·s^−1^) at the end of the experiment (36 DAW).

Similar results were also observed for SPAD readings, with the highest values for the CWoW. It is important to note that the treatments with foliar GB or H_2_O_2_ application began to show higher values compared to the waterlogged control from 18 DAW to the end of the experiment (36 DAW). Finally, the efficiency of PSII (*F_v_/F_m_*) was also affected by the waterlogging conditions, with the greatest negative effects registered at 36 DAW. At this point, the treatments with waterlogging and foliar GB or H_2_O_2_ application had a positive effect on the *F_v_/F_m_* ratio (0.42 for both treatments) compared to CWW (0.28), whereas control plants and without waterlogging showed higher values (0.74) throughout the experiment.

**Figure 2:**
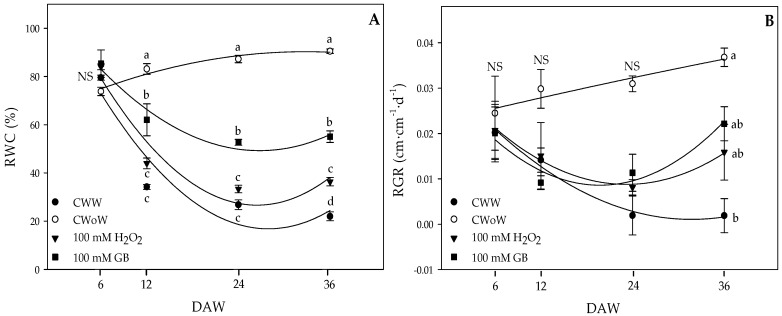
(**A**) Relative Water Content (RWC) and (**B**) Relative Growth Rate (RGR) of Cape gooseberry (*Physalis peruviana* L.) plants ecotype Colombia subjected to two waterlogging periods. Filled circle: control treatment subjected to waterlogging, circle without filling: control treatment without waterlogging, filled triangle: treatment with a concentration of 100 mM of H_2_O_2_, filled square: treatment with a concentration of 100 mM of GB. Evaluated at 6, 12, 24, and 36 days after waterlogging (DAW). Data represent the mean of five plants ± standard error per treatment (*n* = 5). Points followed by different letters indicate statistically significant differences according to the Tukey test (*p* ≤ 0.05).

The Relative Water Content (RWC) showed significant differences between the treatments from 18 DAW (Figure 2A). The best water status throughout the experiment was observed in plants without waterlogging with an RWC of 80%. Therefore, foliar GB or H_2_O_2_ applications favored the RWC of Cape gooseberry plants under waterlogging conditions throughout the experiment. Plants with foliar GB applications showed a higher RWC than waterlogged plants treated with H_2_O_2_ (43.97%) and plants with only waterlogging (34.18%) at 18 DAW. Between 18 and 36 DAW, it was observed that the waterlogging conditions continued to decrease the RWC mainly in the groups of waterlogged plants treated with H_2_O_2_ (36.37%) and CWW (34.18%). Significant differences (*p* ≤ 0.05) were only obtained on the Relative Growth Rate (RGR) (Figure 2B) at 36 DAW. The treatment with foliar GB application at a concentration of 100 mM (0.022 cm) obtained a higher RGR compared to the other plant groups (0.017 cm for control without waterlogging, 0.016 cm for 100 mM, and 0.002 cm for control with waterlogging).

**Figure 3:**
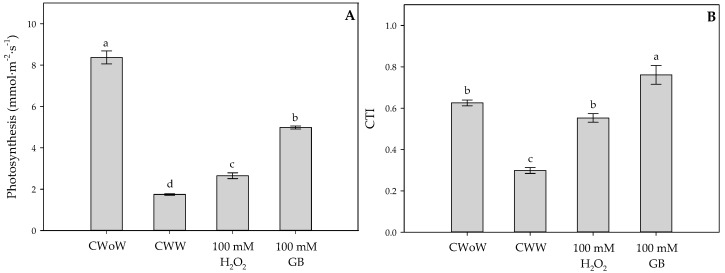
(**A**) Photosynthesis and (**B**) canopy temperature index (CTI) of Cape gooseberry (*Physalis peruviana* L.) plants ecotype Colombia subjected to two waterlogging periods with treatments of control without waterlogging (CWoW), control with waterlogging (CWW), 100 mM of hydrogen peroxide (H_2_O_2_) and 100 mM of glycine betaine (GB). Evaluated at 36 Days After Waterlogging (DAW). Each point represents the mean of the four values. Data represent the average of ten plants ± standard error per treatment (*n* = 5). Bars followed by different letters indicate statistically significant differences according to the Tukey test (*p* ≤ 0.05).

Photosynthesis (Figure 3A) and Canopy Temperature Index (CTI) (Figure 3B) were calculated at 36 DAW. The highest photosynthesis value was obtained in CWoW plants (8.37 mmol·m^−2^·s^−1^). Waterlogging conditions were observed to cause a reduction in the photosynthesis rate of 79% (1.75 mmol·m^−2^·s^−1^). However, photosynthesis under oxygen deficiency in the soil was stimulated by foliar application of GB (4.99 mmol·m^−2^·s^−1^) and H_2_O_2_ (2.65 mmol· m^−2^·s^−1^) (Figure 3A). Similar trends were observed in the CTI where the treatment with foliar GB application (0.76) favored the canopy temperature of Cape gooseberry plants with waterlogging, obtaining higher values than those recorded in plants subjected to waterlogging (0.30) (Figure 3B).

## 3. Discussion

Waterlogging stress causes adverse effects on the physiological and biochemical parameters of cultivated plants [39]. Rao and Li [19] point out that periods of oxygen deficit in the soil greater than 24 h cause a decrease in the leaf gas exchange properties (photosynthesis and stomatal conductance), leaf chlorophyll content, plant growth, and water status. Likewise, stomatal closure and low plant water status caused by waterlogging generate an increase in leaf temperature [40,41]. These responses, induced by waterlogging conditions in the soil, may be associated with physiological dysfunctions, such as impaired water and nutrient uptake caused by a reduction in root hydraulic conductance or root cell death [42,43], restricted CO_2_ entry due to stomatal closure [44,45], low Rubisco activation during CO_2_ assimilation [19], oxidative damage on photosystem II caused by reactive oxygen species (ROS) [44,46], and increased chlorophyllase activity (chlorophyll degradation) and ethylene synthesis [19,47]. High ethylene production in plants under conditions of anoxia or hypoxia is caused by fermentative enzymes (fructose-1,6-bisphosphate aldolase (ALD), enolase (ENO), pyruvate decarboxylase (PDC), and alcohol dehydrogenase 2 (ADH2)) as an adaptive response to oxygen deficit in the soil [48]. Based on the above, the treatment with only waterlogged plants showed physiological affectations such as a reduction of the leaf gas exchange properties, low plant growth, water status, and leaf chlorophyll content, and an increase in leaf temperature in both experiments (Table 1, Table 2, Table 3 and Table 4; Figure 2 and Figure 3). Similar observations have also been reported in Cape gooseberry plants under oxygen deficit conditions in the soil [14,15,25].

Foliar GB or H_2_O_2_ applications (mainly at a dose of 100 mM of each compound) helped to mitigate the negative effects caused by waterlogging conditions in the soil by favoring the evaluated variables in both experiments (Table 1, Table 2, Table 3 and Table 4; Figure 1, Figure 2 and Figure 3). The results obtained in this study confirm similar observations found in other cultivated species in which the exogenously supplied GB and H_2_O_2_ helped to alleviate the negative impact caused by waterlogging conditions on plant physiology. The beneficial role of foliar applications of these compounds on the physiological and biochemical parameters of plants under moderate (approximately 15 days) waterlogging periods has been documented for soybean (exogenous H_2_O_2_ supply also increased leaf gas exchange parameters and growth) [13] and tomato (foliar GB application also enhanced growth and chlorophyll concentration) [28]. Glycine betaine (GB) is a low molecular weight ammonium compound easily absorbed by roots or leaves. It helps to alleviate abiotic stress conditions in plants since it can participate in different physiological processes, such as osmoregulation, stabilization of the quaternary structure of proteins, enzymes (e.g., Rubisco) and membranes, protection of the photosynthetic apparatus, maintenance of the electron flow in the thylakoid membranes and regulation of antioxidant enzymes activity [49,50]. On the other hand, hydrogen peroxide (H_2_O_2_) has a signaling role in the mediation of physiological processes in the plant during the process of acclimatization to different types of abiotic stress. Some of these processes are antioxidant defense, stomatal behavior, regulation of the photosynthesis rate, and promotion of biosynthesis of compatible osmolytes to maintain leaf water content [51,52,53].

Foliar GB and H_2_O_2_ applications favored growth variables (stem length and diameter, leaf area, shoot dry weight, and relative growth rate) and physiological variables such as leaf temperature, stomatal conductance (*g_s_*), maximum photochemical efficiency of PSII (*F_v_/F_m_*), leaf water potential (*Ψ_w__f_*), relative water content (RWC), chlorophyll content, and net photosynthesis (Pn) in Cape gooseberry plants under waterlogging conditions (Table 1, Table 2, Table 3 and Table 4; Figure 2 and Figure 3). It was demonstrated that exogenous applications of H_2_O_2_ increase biomass in cucumber (*Cucumis sativus* L.) plants under water stress conditions compared to plants without H_2_O_2_ [52]. Stem diameter is another important trait to determine the plant's adaptive response and evaluate the efficiency of a treatment to alleviate stress caused by hypoxia conditions in the soil [20,21]. Soybean (*Glycine max* L.) seeds treated exogenously with H_2_O_2_ increased the stem diameter of seedlings exposed to a prolonged waterlogging period (32 days) compared to seedlings under conditions of oxygen deficit in the soil and without the application of this compound [13]. It has also been confirmed that exogenously supplied H_2_O_2_ stimulates aerenchyma formation in plants, such as rice [54].

In this study, it was observed that foliar H_2_O_2_ sprays increase stem diameter by promoting lysigenous aerenchyma (internal gas space) formation for adaptation to waterlogging conditions [54]. Finally, the positive effect of foliar GB applications on growth parameters was also reported in tomato (*Solanum lycopersicum* L.) plants favoring dry matter accumulation in the shoot and root under waterlogging conditions [28]. 

An increase in leaf gas exchange parameters (Pn and *g_s_*), chlorophyll content, *F_v_/F_m_* and plant water relations (*Ψ_w__f_* and RWC) after exogenous application of H_2_O_2_ and GB has also been reported by several authors under abiotic stress conditions. For example, Andrade et al. [13] showed that exogenous H_2_O_2_ applications increased the shoot and root dry matter, root volume, stem diameter, Pn, *g_s_*, transpiration, leaf chlorophyll content and activity of antioxidative enzymes in soybean plants under waterlogging stress. On the other hand, Sorwong and Sakhonwasee [55] also observed a positive effect on leaf gas exchange parameters (Pn and *g_s_*) and *F_v_/F_m_* ratio in Mexican marigold (*Tagetes erecta* L.) plants subjected to abiotic stress after foliar treatment with GB. Likewise, GB application caused a significant increase in the content of photosynthetic pigments (chlorophyll and carotenoids) in tomato plants exposed to conditions of oxygen deficiency in the soil [28]. 

In this study, H_2_O_2_ was found to mitigate the negative effects of waterlogging by improving plant growth. This response may be associated with the fact that H_2_O_2_ can activate signaling pathways to stimulate cell proliferation and also increase leaf area [56], cell differentiation [57], and plant elongation [58]. Additionally, this compound enhanced the gas exchange parameters, efficiency of PSII, and leaf chlorophyll content in plants under conditions of oxygen deficit in the soil. The positive results of exogenous H_2_O_2_ application could be related to the induction of plant tolerance to different abiotic stress conditions by modulating processes involved in ROS detoxification, the increase in glutathione (GSH) and ascorbate (AsA) contents, the biosynthesis of proline to protect the photosynthetic machinery, and the regulation of stomatal conductance [13,59]. Finally, H_2_O_2_ sprays favored the accumulation of compatible solutes such as proline, maintaining the leaf RWC [51,52]. 

Applications of GB to Cape gooseberry plants under waterlogging conditions showed a biostimulant effect on plant growth and the evaluated physiological parameters. It has been reported that foliar GB sprays can quickly penetrate the leaf surface and be easily transported to other plant organs, where it would contribute to improving tolerance to different types of abiotic stress [60]. This compound may be involved in the inhibition of ROS accumulation, protection of photosynthetic machinery, accumulation of compatible solutes to maintain turgidity in cells, activation of some stress-related genes, and protection of the cell membrane and quaternary structure of proteins [61,62]. Likewise, GB regulates stomatal movements by prolonging their opening under a condition of abiotic stress as a consequence of increased osmoprotective activity [63]. Finally, an increase in the activity of antioxidant enzymes such as catalases (CAT) and peroxidases (POD) has been reported in response to GB application by regulating the oxidative stress generated by waterlogging conditions in the soil [13]. Leaf temperature has been used as an indicator of stress in plants [64]. In the present study, exogenous H_2_O_2_ and GB applications favored leaf temperature regulation by improving the CTI (Figure 3 and Table 2). As mentioned above, these molecules have an osmoprotective or stomatal regulation effect that directly favors the plant water status, resulting in lower leaf temperature. Finally, it was observed that WTC and CTI can be associated with physiological variables such as leaf gas exchange properties or plant growth, which can be considered to evaluate the effectiveness of treatments to mitigate the effects of waterlogging [65].

The most inexpensive way to deal with short-term waterlogging periods is improving plant tolerance to low oxygen conditions in the soil [66]. In recent years, studies have focused on the evaluation of the physiological and biochemical responses to different periods of waterlogging, finding that Cape gooseberry plants are highly susceptible to short-term periods of this stress [14,15,67]. In this regard, the analysis of easy-to-use, economic, and profitable techniques is necessary to mitigate the effects of short or intermittent periods of waterlogging. It has been observed that the use of foliar applications of organic compounds or elicitors is an important tool to help plants acquire tolerance to short periods of waterlogging [19,66]. The results of present study demonstrated that exogenously supplied GB or H_2_O_2_ may be an appropriate agronomic practice to counteract the negative effects of non-prolonged waterlogging periods, since these treatments increased the tolerance of a susceptible plant (expressed as WTI) such as Cape gooseberry. Exogenous application of elicitors (hormones) or organic compounds (botanical extracts) have also favored plant tolerance to a short period of stress [25]. However, the novelty of this research was that foliar application of the evaluated compounds increased Cape gooseberry tolerance to intermittent periods of waterlogging. 

There is still a lack of knowledge to understand the advantages or disadvantages of several techniques to manage Andean fruit crops under different waterlogging periods. The obtained results indicate a series of advantages from the physiological and crop management points of view; it is important to highlight that the exogenous application of organic compounds or elicitors is a technique that can help plants to cope with the unfavorable condition of waterlogging relatively fast when soil conditions are limiting. These observations are in agreement with other studies that report the foliar application of organic or nitrogenous compounds, elicitors, or phytohormones as an efficient technique to reduce the negative effects of short or moderate periods of waterlogging [19,20,25]. Another advantage of this study is that the analysis of responses such as growth, stem diameter, plant water status, leaf gas exchange, and plant temperature has recently helped to understand the acclimatization mechanisms of plants of the *Solanaceae* family, mainly Andean fruit trees, to conditions of oxygen deprivation in the soil in tropical countries. The obtained information has provided support for the evaluation and development of efficient crop management techniques [21,68,69]. However, it has been reported that foliar applications of compounds such as growth regulators, nutrients, or elicitors are not effective for prolonged periods of waterlogging. Thus, the combination of techniques such as soil drainage and crop management practices (foliar applications) is still required [66].

## 4. Materials and Methods

### 4.1. Plant Material and General Growth Conditions of the Experiments

Two experiments were carried out separately between March and July 2019 under greenhouse conditions at the Faculty of Agricultural Sciences of the Universidad Nacional de Colombia, (4°35′56″ and 74°04′51″), Bogotá campus. The general growth conditions in the greenhouse in both experiments were: average temperature of 25/15 °C, 60%–80% relative humidity, and a natural photoperiod of 12 h. For both experiments, 2-month-old Cape gooseberry (*Physalis peruviana* L.) ecotype Colombia seedlings were transplanted into 2 L capacity plastic pots with a mixture of peat and sand (3:1 *v/v*) as substrate. The plants were watered every day from the transplant until the beginning of treatments with 50 mL of a nutritive solution prepared with a complete liquid fertilizer (Nutriponic^®^, Walco SA, Colombia) at a concentration of 5 mL·L^−1^ H_2_O. The final concentration of the nutrient solution was as follows: 2.08 mM Ca (NO_3_)_2_·4H_2_O, 1.99 mM MgSO_4_·7 H_2_O, 2.00 mM NH_4_H_2_PO_4_, 10.09 mM KNO_3_, 46.26 nM H_3_BO_3_, 0.45 nM Na_2_MoO_4_·2H_2_O, 0.32 nM CuSO_4_·5H_2_O, 9.19 nM MnCl_2_·4H_2_O, 0.76 nM ZnSO_4_·7H_2_O, and 19.75 nM FeSO_4_·H_2_O. The volume of irrigated water was obtained using the technique described by Hainaut et al. [70], in which daily evapotranspiration was estimated gravimetrically. The treatments in each experiment were established from 45 days after transplanting (DAT) and these are detailed below: 

#### 4.1.1. Experiment 1—Evaluation of Different Glycine Betaine (GB) or Hydrogen Peroxide Doses (H_2_O_2_) under a Waterlogging Period

At 45 DAT, when plants reached five fully expanded leaves, eight treatment groups were established to estimate the effect of foliar GB and H_2_O_2_ sprays under waterlogging conditions. The treatment groups were as follows: (1) plants without waterlogging and with no GB or H_2_O_2_ application (absolute control), (2) waterlogged plants with no GB or H_2_O_2_ application, (waterlogged control), (3) waterlogged plants with foliar H_2_O_2_ sprays at concentrations of 25, 50 or 100 mM (JGB SA, Cali, Colombia), (4) waterlogged plants with foliar GB sprays at concentrations of 25, 50 or 100 mM (VivaGrow, Westminster, CO, USA). Waterlogging treatments consisted in placing the plants in plastic containers with dimensions of 53 × 53 × 30 cm and a capacity of 120 L, filled until reaching a 5 cm water level on the root neck. The plants were subjected to a 6-day waterlogging period (between 45 and 51 DAT) since previous studies showed that this period caused damage to Cape gooseberry plants [14,15,67]. Foliar GB or H_2_O_2_ applications were performed at 0, 6, and 10 DAW using an application volume of 20 mL, wetting both the upper and lower surfaces of leaves using a 1.8 L manual spray pump (Royal Condor Garden^®^, Soacha, Colombia). All foliar applications were carried out between 07:00 and 09:00 h (with sunrise at 06:00 h). Each treatment group consisted of 10 plants (five replicates per sampling point), for a total of 80 plants in this experiment, which were arranged in a completely randomized design (CRD) in the greenhouse. Finally, the experiment lasted approximately 60 days.

#### 4.1.2. Experiment 2—Evaluation of the Most Efficient Doses of Glycine Betaine (GB) and Hydrogen Peroxide (H_2_O_2_) (Experiment 1) in Plants Exposed to Two Waterlogging Periods

Based on the first trial, the dose of the two chemical compounds (GB and H_2_O_2_) that showed the best response to mitigate the waterlogging stress was selected (Figure 1). Four groups of treatments were also established when plants reached five fully mature leaves at 45 DAT. The treatments are described as follows: (1) plants without waterlogging and with no GB or H_2_O_2_ sprays (absolute control), (2) waterlogged plants with no GB or H_2_O_2_ sprays (waterlogged control), (3) waterlogged plants with foliar H_2_O_2_ sprays at a concentration of 100 mM, and (4) waterlogged plants with foliar GB sprays at a concentration of 100 mM. Waterlogging treatments were also carried out by placing the plants in plastic containers with dimensions of 53 × 53 × 30 cm and a capacity of 120 L, which were filled until reaching a water level of 5 cm above the root neck. In this experiment, plants were subjected to two different waterlogging periods to quantify the effect of chemicals on mitigation under two short time (6 days) stress conditions. The first stress period was established between 45 and 51 DAT, while the second period was between 63 and 69 DAT. Between each waterlogging period, the plants were removed from each plastic container to allow water to drain until reaching the field capacity of the substrate. Subsequently, plants were watered during the recovery period (between 52 and 62 DAT) according to the evapotranspiration rate for 12 days. Foliar GB or H_2_O_2_ applications were also performed at 0, 3, 6, and 9 DAW between 07:00 and 09:00 h, wetting the upper and lower surfaces of leaves using the manual spray pump. Each treatment group consisted of 20 plants (five replicates per sampling point), for a total of 80 plants in this experiment, which were arranged in a completely randomized design (CRD) in the greenhouse. Finally, the experiment lasted 85 days.

### 4.2. Stomatal Conductance, Relative Chlorophyll Content, and Efficiency of PSII (F_v_/F_m_)

Stomatal conductance (*gs*) was estimated using a portable porometer (SC-1, Decagon Devices Inc., Pullman, WA, USA) between 10:00 and 13:00 h on a fully expanded leaf in the middle portion of the canopy. The relative chlorophyll content was then measured with a chlorophyll meter (AtLeaf, FT Green LLC Wilmington, USA), also on the same leaves used for *gs* readings in both experiments. Finally, the leaves used for *gs* and SPAD readings were dark-adapted with clips for 20 min to determine the maximum efficiency of PSII (*F_v_/F_m_*) by using a modulated fluorometer (MINI-PAM, Walz, Effeltrich, Germany) with an actinic light pulse of up to 2600 µmol·m^−2^·s^−1^ on their surface.

### 4.3. Plant Growth Parameters (Stem Diameter and Height, Leaf Area, Shoot Dry Weights and Relative Growth Rate)

Plant height and root neck diameter were recorded weekly with a ruler and vernier caliper, respectively. Then plants were harvested and separated into each of the shoot organs (leaves and stems). The leaf area of each plant was estimated by taking a photograph of the leaves of the plant canopy and, subsequently, the digital images were analyzed with a Java image-processing program (Image J; National Institute of Mental Health, Bethesda, MD, USA). Finally, the harvested organs were dried for 72 h in an oven at 70 °C to determine their respective dry weight (DW). In general, measurements of the above variables were performed at 4 and 13 DAW for experiment 1, and at 6, 18, 24, and 36 DAW for experiment 2, respectively.

On the other hand, RGR was determined for experiment 2. It was indirectly calculated using the length of the stem regarding the different sampling days. RGR was calculated using the following Equation (1):

(1) $$\mathrm{RGR}=\frac{\mathrm{Ln}\;SL\;T2-\mathrm{Ln}\;SL\;T1}{\mathrm{DS}2-\mathrm{DS}1}$$

where *SL T*2 means stem length at time 2, *SL T*1 means stem length at time 1, DS2—DS1 means the difference in the number of days between sample 2 and sample 1.

### 4.4. Leaf Temperature and Canopy Temperature Index (CTI)

In experiment 1, the same leaves used for *gs*, leaf relative chlorophyll content, and *F_v_/F_m_* readings were also selected to determine leaf temperature using an infrared thermometer (Cole Parmer Instruments, Vernon Hills, IL 60061, USA). The readings were taken at 4 and 13 DAW.

In experiment 2, CTI was determined by means of a thermal camera (FLIR C2, FLIR Systems, Wilsonville, OR, USA), using Equation (2) described by Jones [71]:

(2) $$\mathrm{CTI}=\frac{\mathrm{PT}-\mathrm{Twl}}{\mathrm{Tdl}-\mathrm{Twl}}$$

where PT is the plant temperature, Twl is the temperature of the wet leaf, and Tdl is the temperature of the dry leaf. PT is determined by taking a thermal photograph at a distance of 0.9 m from the entire plant. Likewise, Tdl was estimated by collecting a leaf and Twl was obtained by wetting the leaf with a mixture of water and an agricultural adjuvant (Agrotin, Bayer CropScience, Bogotá, Colombia). Both leaves were placed on a white Styrofoam surface at the base of the plant pot. Plant canopy and reference leaves (Tdl and Twl) temperatures were analyzed using the software FLIR^®^ Tools Plus 3.1.13080.1002 (FLIR^®^ Systems, Wilsonville, OR, US). Thermal images were taken at noon at 36 DAW.

### 4.5. Water Relations (Leaf Water Potential and Relative Water Content (RWC))

After estimating the leaf temperature in experiment 1, the leaves were cut at the petiole with a scalpel to immediately record the leaf water potential (*Ψ_wf_*) with a Scholander pressure chamber (PMS Instruments, Albany, OR, USA). The chamber was then sealed and gradually pressurized with nitrogen. The water potential was recorded by observing the expulsion of the sap from the xylem system out of the cut edge of the leaf petiole with an *X*15 magnifying glass. The *Ψ_wf_* was obtained at noon at 4 and 13 DAW. In experiment 2, a leaf was taken from the middle third of the plant to determine the (RWC). Five 25 mm diameter discs were cut and their fresh weight (FW) was obtained. Subsequently, the discs were placed in a Petri dish with water for 24 h at laboratory temperature to determine the turgid weight (TW). Finally, the discs were dried for 72 h in an oven at 70 °C and their DW was determined. The RWC was calculated at 6, 18, 24, and 36 DAW using the following Equation (3):

(3) $$\mathrm{RWC}=\frac{\left(\mathrm{FW}-\mathrm{DW}\right)}{\left(\mathrm{TW}-\mathrm{DW}\right)}\times 100$$

### 4.6. Waterlogging Tolerance Coefficient (WTC)

The WTC was indirectly calculated to determine the tolerance of treatments to waterlogging, using the shoot dry weight of the waterlogged treatments in relation to the control treatment without waterlogging [65]. The WTC was obtained at 13 DAW in experiment 1 using the following Equation (4):

(4) $$\mathrm{WTC}=\frac{{\mathrm{DW}}_{\mathrm{WT}}}{{\mathrm{DW}}_{\mathrm{CT}}}$$

where DW_WT_ is the shoot dry weight of waterlogged treatments with or without foliar GB or H_2_O_2_ sprays, whereas DW_CT_ is the shoot dry weight of the absolute control treatment (plants without waterlogging and foliar sprays).

### 4.7. Photosynthesis

Photosynthesis was estimated using a portable photosynthesis meter (LICOR 6200, Lincoln, NE, USA) on a leaf from the middle third of the plant in experiment 2. Measurements were taken on completely sunny days between 11:00 and 13:00 h at 36 DAW. During photosynthesis measurements, the conditions inside the chamber were as follows: photosynthetically active radiation (PAR) greater than 800 μmol m^−2^·s^−1^, leaf temperature of 27 ± 5 °C, and leaf to air water vapor pressure difference of 1.8 ± 0.5 kPa.

### 4.8. Experimental Design and Data Analysis

The data obtained from the first and second experiments were analyzed using a completely randomized design (CRD) in which each treatment had five plants as replicates. All percentage values were transformed using the arcsine transformation before analysis. Likewise, a correlation analysis was performed between the WTC and the leaf area to determine the best dose of the products used in experiment 1. When the analysis of variance (ANOVA) showed significant differences (*p* ≤ 0.05), a Tukey post hoc test was used for mean comparison. The data were analyzed with the Statistix v 9.0 software (Analytical Software, Tallahassee, FL, USA) and the graphs were developed in SigmaPlot 12.0 (Systat Software, San Jose, CA, USA).

## 5. Conclusions

In summary, the present study continued to show the susceptibility of Cape gooseberry plants to waterlogging conditions, since this plant species shows a reduction in growth mainly associated with a low photosynthetic rate and water status under conditions of oxygen deficiency in the soil. However, foliar H_2_O_2_ or GB applications at a concentration of 100 mM helped to lessen the waterlogging conditions on Cape gooseberry plants and favored their physiological response. The foregoing allows us to conclude that the use of H_2_O_2_ or GB can be a viable tool in managing stress conditions due to moderate waterlogging in Cape gooseberry crops when periods of heavy rainfall are expected.

## Figures and Tables

**Table 1:** Stem length, stem diameter, leaf area, and shoot dry weight of Cape gooseberry (*Physalis peruviana* L.) plants ecotype Colombia subjected to a waterlogging period and with exogenous applications of 25, 50, and 100 mM of hydrogen peroxide (H_2_O_2_) or glycine betaine (GB), respectively. Control without waterlogging (CWoW) and control with waterlogging (CWW). Evaluated at 4 and 13 days after waterlogging (DAW).

Treatment	4 DAW				13 DAW			
	Stem Length (cm)	Stem Diameter (cm)	Leaf Area (cm^2^)	Shoot Dry Weight (g)	Stem Length (cm)	Stem Diameter (cm)	Leaf Area (cm^2^)	Shoot Dry Weight (g)
CWoW	24.62 a ^1^	0.664 a	369.74 a	1.45 a	25.50 a	0.72 a	376.58 a	2.06 a
CWW	16.70 c	0.428 b	156.37 d	0.48 e	16.56 d	0.42 c	116.14 d	0.39 d
Waterlogging + 25 mM H_2_O_2_	20.82 abc	0.524 ab	254.84 c	0.65 de	18.10 cd	0.55 bc	139.02 cd	0.57 cd
Waterlogging + 50 mM H_2_O_2_	19.50 bc	0.500 b	255.60 c	0.64 de	19.30 c	0.50 bc	166.86 c	0.65 cd
Waterlogging + 100 mM H_2_O_2_	22.40 ab	0.556 ab	311.18 b	0.99 c	22.10 b	0.55 b	171.18 c	0.71 c
Waterlogging + 25 mM GB	22.60 ab	0.470 b	240.54 c	0.74 d	18.40 cd	0.49 bc	152.78 c	0.67 cd
Waterlogging + 50 mM GB	19.98 bc	0.490 b	266.36 c	1.00 c	18.84 cd	0.52 bc	169.98 c	0.69 cd
Waterlogging + 100 mM GB	20.90 ab	0.484 b	318.51 b	1.25 b	18.20 cd	0.53 bc	222.56 b	1.06 b
Significance (*p* value)	0.0001	0.0013	0.0000	0.0000	0.0000	0.0000	0.0000	0.0000
CV (%) ^2^	9.67	14.45	6.35	9.26	5.99	12.17	9.33	18.07

^1^ Values (*n* = 5) within a column followed by different letters are significantly different from *p* ≤ 0.05 according to the Tukey test. ^2^ CV: Coefficient of variation.

**Table 2:** Estimation of physiological parameters: Leaf temperature, stomatal conductance, efficiency of photosystem II (PSII) (*Fv/Fm*), and leaf water potential in Cape gooseberry (*Physalis peruviana* L.) plants ecotype Colombia subjected to a waterlogging period with exogenous applications of 25, 50, and 100 mM of hydrogen peroxide (H_2_O_2_) or glycine betaine (GB), respectively. Control without waterlogging (CWoW) and control with waterlogging (CWW). Evaluated at 4 and 13 Days After Waterlogging (DAW).

Treatment			4 DAW				13 DAW	
	Leaf Temperature (°C)	Stomatal Conductance (mmol CO_2_·m^−2^·s^−1^)	Efficiency of PSII (*F_v_/F_m_*)	Leaf Water Potential (−Mpa)	Leaf Temperature (°C)	Stomatal Conductance (mmol CO_2_·m^−2^·s^−1^)	Efficiency of PSII (*F_v_/F_m_*)	Leaf Water Potential (−Mpa)
CWoW	18.85 a ^1^	194.42 a	0.82 a	0.23 a	18.93 a	190.26 a	0.81 ab	0.30 a
CWW	26.89 d	157.10 c	0.57 c	0.54 c	28.49 d	42.76 d	0.64 ab	0.66 c
Waterlogging + 25 mM H_2_O_2_	24.52 c	189.66 a	0.63 bc	0.49 bc	26.98 cd	66.16 c	0.77 ab	0.62 c
Waterlogging + 50 mM H_2_O_2_	24.16 c	149.00 b	0.70 abc	0.45 bc	26.92 cd	93.40 b	0.84 a	0.59 bc
Waterlogging + 100 mM H_2_O_2_	24.71 c	191.78 a	0.70 abc	0.420 bc	24.16 b	94.56 b	0.78 ab	0.53 bc
Waterlogging + 25 mM GB	22.12 b	176.78 b	0.74 ab	0.49 bc	26.49 c	66.62 c	0.63 b	0.54 bc
Waterlogging + 50 mM GB	23.87 c	156.90 b	0.81 a	0.48 bc	26.49 c	93.36 b	0.74 ab	0.57 bc
Waterlogging + 100 mM GB	22.27 b	180.70 a	0.80 a	0.37 ab	24.07 b	95.64 b	0.75 ab	0.45 ab
Significance (*p* value)	0.0000	0.0000	0.0000	0.0001	0.0000	0.0000	0.0270	0.0175
CV (%) ^2^	1.86	6.52	4.67	18.74	3.52	10.96	8.6	18.97

^1^ Values (*n* = 5) within a column followed by different letters are significantly different from *p* ≤ 0.05 according to the Tukey test. ^2^ CV: Coefficient of variation.

**Table 3:** Growth parameters (leaf area, shoot dry weight and stem diameter) of Cape gooseberry (*Physalis peruviana* L.) plants ecotype Colombia subjected to two waterlogging periods with control treatments without waterlogging (CWoW), control with waterlogging (CWW), 100 mM of hydrogen peroxide (H_2_O_2_) and 100 mM of glycine betaine (GB). Evaluated at 6, 18, 24, and 36 days after waterlogging (DAW).

Treatment	Stem Diameter (cm)				Shoot Dry Weight (g)				Foliar Area (cm^2^)			
	DAW				DAW				DAW			
	6	18	24	36	6	18	24	36	6	18	24	36
CWoW	0.29 a ^1^	0.34 a	0.43 a	0.49 a	2.61 a	6.14 a	5.95 a	9.90 a	584.7 a	882.2 a	1163.2 a	1236.1 a
CWW	0.21 b	0.24 c	0.29 c	0.31 c	0.93 b	2.22 b	2.17 b	1.59 b	104.2 c	100.9 b	106.8 c	25.8 c
Waterlogging + 100 mM H_2_O_2_	0.25 ab	0.27 bc	0.32 bc	0.35 b	1.53 ab	2.02 b	2.83 b	1.99 b	154.3 b	142.1 b	202.4 bc	91.5 b
Waterlogging + 100 mM GB	0.30 a	0.31 ab	0.34 b	0.39 b	2.21 ab	2.33 b	3.06 b	1.75 b	135.4 ab	129.8 b	221.9 b	95.5 b
Significance (*p* value)	0.006	0.000	0.000	0.000	0.046	0.000	0.000	0.000	0.000	0.000	0.000	0.000
CV (%) ^2^	11.69	6.56	6.12	5.53	43.11	20.71	22.76	60.06	6.41	15.14	12.08	7.84

^1^ Values (*n* = 5) within a column followed by different letters are significantly different from *p* ≤ 0.05 according to the Tukey test. ^2^ CV: Coefficient of variation.

**Table 4:** Physiological parameters (stomatal conductance, soil plant analysis development (SPAD) chlorophylls, and efficiency of PSII) of Cape gooseberry (*Physalis peruviana* L.) plants ecotype Colombia subjected to two waterlogging periods with treatments of control without waterlogging (CWoW), control with waterlogging (CWW), 100 mM of hydrogen peroxide (H_2_O_2_) and 100 mM of glycine betaine (GB). Evaluated at 6, 18, 24, and 36 days after waterlogging (DAW).

Treatment	Stomatal Conductance (mmol CO_2_·m^−2^·s^−1^)				SPAD Chlorophylls				Efficiency of PSII (*F_v_/F_m_*)			
	DAW				DAW				DAW			
	6	18	24	36	6	18	24	36	6	18	24	36
CWoW	195.8 a ^1^	129.1 a	215.8 a	117.7 a	38.3 a	32.8 a	36.5 a	42.2 a	0.83 a	0.82 a	0.75 a	0.74 a
CWW	18.9 b	14.1 b	13.9 b	9.90 c	31.3 ab	19.6 b	18.7 b	11.0 c	0.67 ab	0.68 a	0.58 a	0.28 c
Waterlogging + 100 mM H_2_O_2_	27.6 b	24.9 b	29.1 b	40.6 b	33.6 ab	23.6 ab	24.7 ab	17.5 b	0.65 b	0.77 a	0.45 a	0.42 b
Waterlogging + 100 mM GB	32.4 b	20.6 b	33.2 b	31.8 b	29.8 b	23.1 ab	28.6 ab	12.0 bc	0.78 ab	0.73 a	0.69 a	0.42 b
Significance (*p* value)	0.000	0.000	0.000	0.000	0.020	0.012	0.008	0.000	0.025	0.099	0.154	0.000
CV (%) ^2^	16.53	46.02	22.3	12.94	10.25	19.28	22.04	13.75	10.47	9.74	30.14	11.69

^1^ Values (*n* = 5) within a column followed by different letters are significantly different from *p* ≤ 0.05 according to the Tukey test. ^2^ CV: Coefficient of variation.
